# Combined transhepatic and transjugular approach for mechanical thrombectomy of massive TIPS thrombosis

**DOI:** 10.1016/j.radcr.2022.01.086

**Published:** 2022-03-02

**Authors:** Jack B. Newcomer, Emad A. Chishti, Driss Raissi

**Affiliations:** a Department of Radiology, University of Kentucky College of Medicine, Lexington, Kentucky, 40506, USA; b Division of Vascular and Interventional Radiology, Department of Radiology, University of Kentucky College of Medicine, Lexington, Kentucky, 40506, USA

**Keywords:** TIPS, Transjugular intrahepatic portosystemic shunt, Thrombosis, Portal hypertension, Thrombectomy, Thrombolysis

## Abstract

Transjugular intrahepatic portosystemic shunt (TIPS) is a well-validated decompressive therapy option to manage ascites and variceal bleeding secondary to portal hypertension. Complications following TIPS procedures include hepatic encephalopathy, liver failure, and TIPS dysfunction. TIPS dysfunction is due to occlusion or stenosis of the TIPS shunt and can be caused by acute or chronic thrombosis. TIPS thrombosis is often treated with mechanical thrombectomy or catheter-directed thrombolytic therapy. Most cases of in-stent occlusion can be treated via a transjugular approach with recanalization or placement of additional stents. We present a case of a 72-year-old female who presented with worsening ascites 17 months after initial TIPS procedure; she was found to have a large thrombus completely occluding the TIPS stent. In our case, a combined transhepatic and transjugular approach was required for TIPS revision given the extent of well-organized clot located near the hepatic venous end of the stent, resulting from prolonged stent occlusion. This was an extremely challenging scenario with two overlapping covered stents and a bare metal stent at the hepatic venous end in the setting of chronic thrombosis and a well-organized fibrous cap. The case highlights the need for optimal initial placement of the primary TIPS shunt to avoid the need for subsequent complex interventions to maintain TIPS shunt patency.

## Introduction

Transjugular intrahepatic portosystemic shunt (TIPS) is a well-validated decompressive therapy option to manage ascites and variceal bleeding secondary to portal hypertension [1]. Complications following TIPS procedures include hepatic encephalopathy, liver failure, infection, and TIPS dysfunction. TIPS dysfunction is due to occlusion or stenosis of the shunt and can be caused by acute of chronic thrombosis [2]. Patients with TIPS dysfunction can present with recurrent variceal bleeding or worsening ascites. The majority of patients who undergo TIPS procedures will require reintervention to maintain patency [3].

The incidence of TIPS thrombosis has decreased significantly with the widespread use of polytetrafluroethylene stents instead of bare metal stents [4,5]. However, TIPS stenosis can still occur over time and predisposes patients to thrombosis with resultant occlusion. Most patients with TIPS occlusion have an underlying stenosis, with the hepatic vein being the most common site of pathology followed by in-stent stenosis. TIPS dysfunction related to thrombosis can be treated with mechanical thrombectomy or catheter-directed thrombolytic therapy. Treatment options for underlying stenosis include placement of additional stents and/or angioplasty. In some cases, placement of a second parallel TIPS stent can also be performed [6].

We present a case of a 72-year-old-female who presented for a TIPS revision and was found to have a large thrombus completely occluding the TIPS stent. She was treated via a combined transjugular and transhepatic approach to cannulate the TIPS stent, with subsequent thrombectomy performed due to the large clot burden.

## Case report

**Fig. 1:**
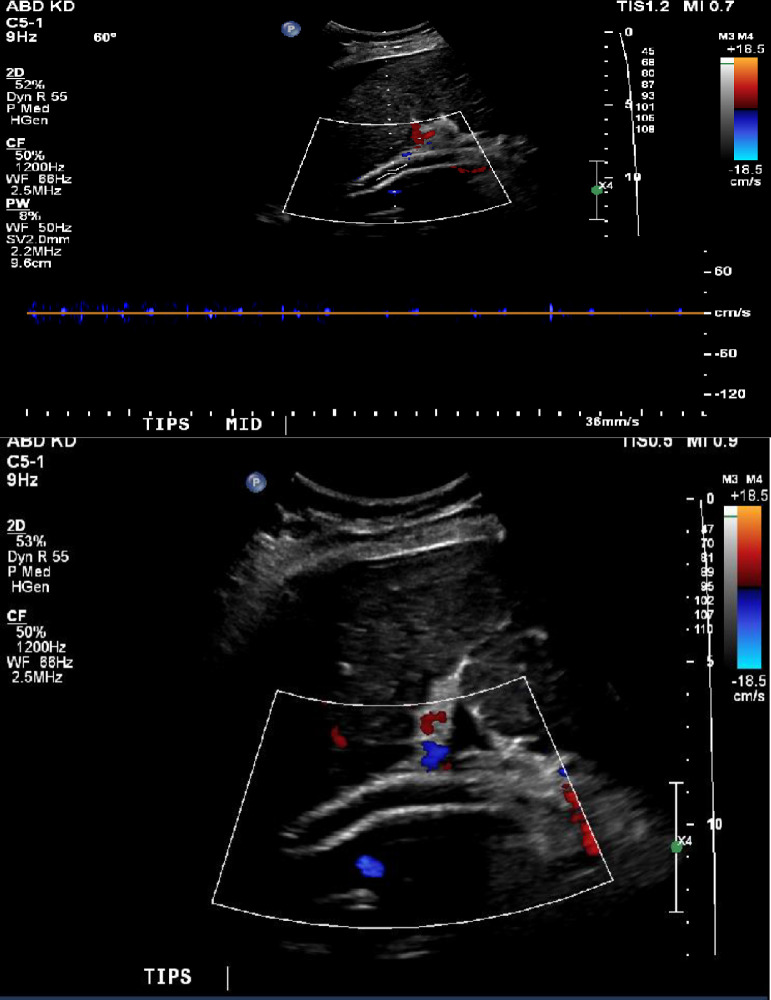
Duplex ultrasound images of the TIPS stent showing no blood flow within the TIPS shunt.

**Fig. 2:**
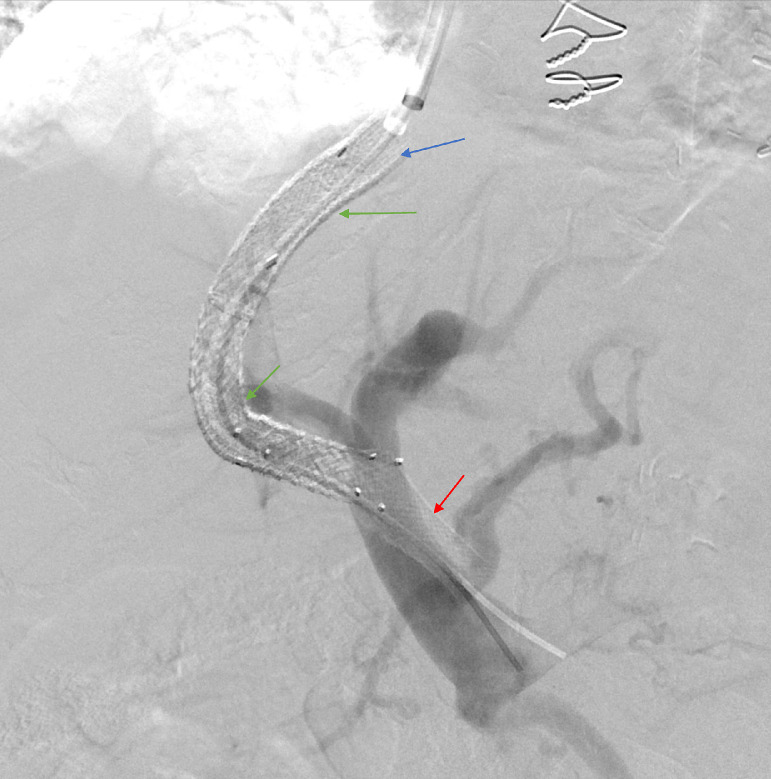
Portal angiography at an outside hospital showing occluded TIPS, patent main portal venous system, 2 covered stents (green arrow), one extension uncovered stent (blue arrow) and a malpositioned portal end stent (red arrow).

A 72-year-old female with a past medical history of cirrhosis secondary to non-alcoholic steatohepatitis, esophageal varices status-post multiple band ligations, bilateral carotid endarterectomy, cerebrovascular accident, and coronary artery bypass grafting presented to our tertiary medical center for TIPS revision 17 months after initial placement. She underwent initial TIPS shunt placement for management of persistent ascites requiring large volume paracentesis twice per week. After this, her paracentesis burden was reduced to once per week, however, at 13 months following initial TIPS, she once again began to require large volume paracentesis twice per week. TIPS revision was then performed by means of TIPS stent angioplasty and placement of an extension stent at the portal venous end, and two additional stents at the hepatic venous end, one covered and one uncovered. However, the patient experienced only minimal improvement in symptoms prompting further evaluation at our institution. Both the initial TIPS procedure and first revision were performed at an outside hospital. Doppler ultrasound 3 months prior to the revision described in this report showed an occluded TIPS without significant flow and thrombus extending into the superior mesenteric vein. (Fig. 1). The patient was known to have an occluded TIPS from outside hospital records for over 10 months, with portal angiogram showing the occlusion (Fig. 2).

**Fig. 3:**
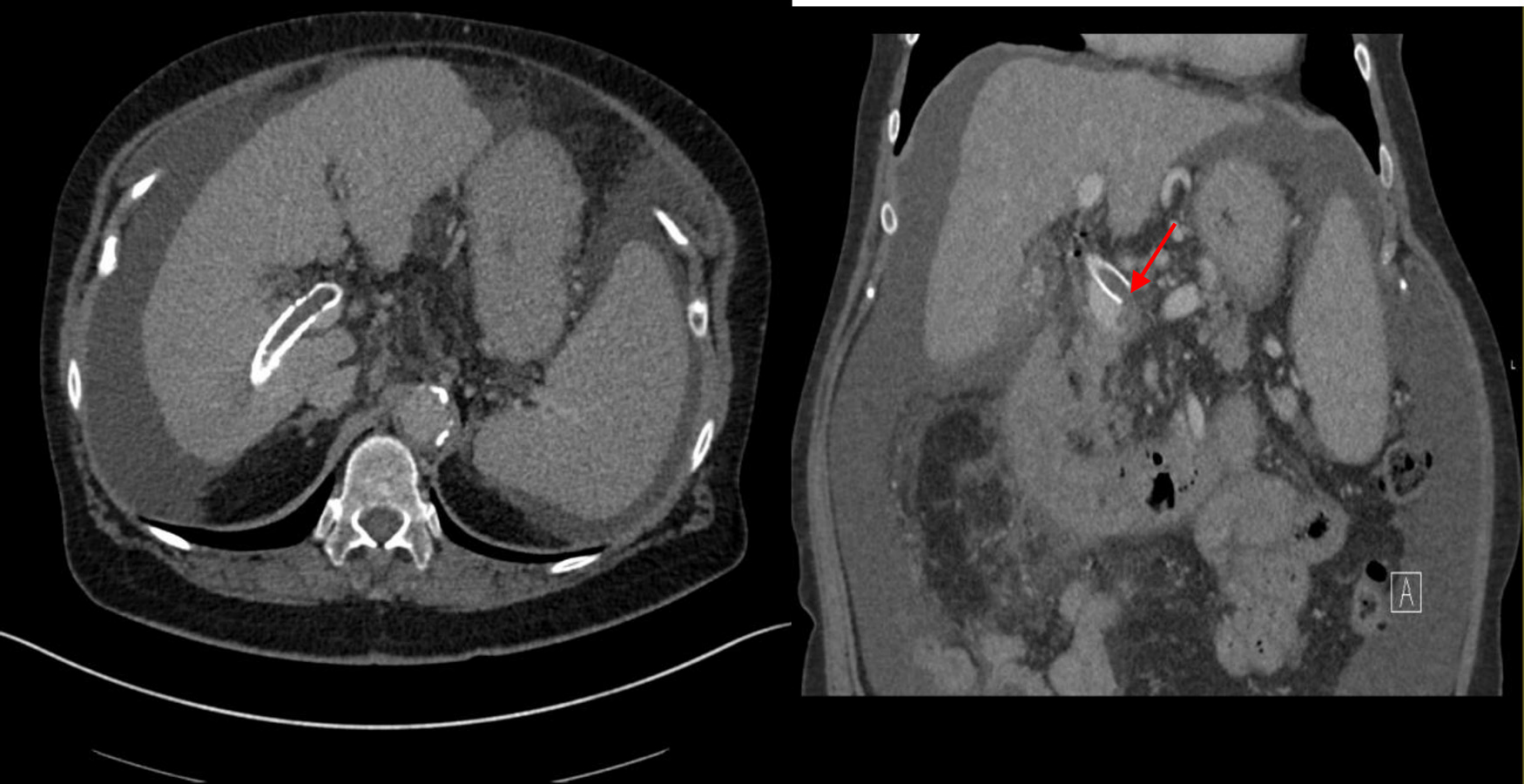
Venous phase of a contrast-enhanced CT of the abdomen showing thrombus within the TIPS stent extending to the superior mesenteric vein inflow. Both axial (Fig. 3A) and coronal images (Fig. 3B) are shown.

**Fig. 4:**
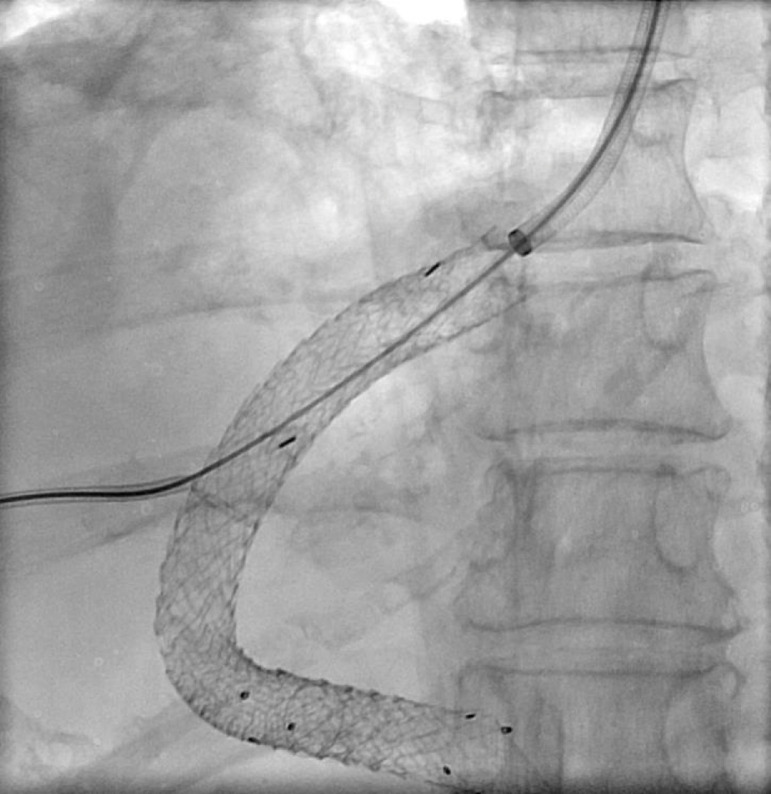
Spot fluoroscopy image showing “flossed” access after combined transhepatic and transjugular approaches, to perform thrombectomy within the mesenteric venous system.

Contrast-enhanced CT abdomen performed at our hospital showed a thrombus within the TIPS stent and our interventional radiology team was consulted for TIPS revision (Fig. 3). The right internal jugular vein was accessed using Seldinger technique under ultrasound guidance. Initial attempts to cannulate the TIPS stent from transjugular route were unsuccessful. Sharp recanalization attempts of the hepatic venous end of the stent using the 16-gauge TIPS kit needle were also unsuccessful. At this point, cannulation of the TIPS stent percutaneously via a transhepatic approach was attempted. Using a 22-Fr Chiba needle (Cook Medical, Bloomington, Indiana, USA), the upper third of the TIPS stent was accessed using the triangulation method under fluoroscopic guidance, however, 22-gauge Chiba needle (Cook Medical, Bloomington, IN) would not advance through the stent wall, likely due to the existence of 2 overlapping TIPS stents. We then decided to use an 18-gauge Hawkins needle (Cook Medical, Bloomington, IN), and multiple attempts were made to pass various types of .018 microwires (Nitinol wire, V18, and Nitrex wire) [Medtronic, Minneapolis, MN], and finally a .035-inch stiff Glidewire through the TIPS stent. However, extreme resistance was experienced while at the hepatic venous end, likely due to a chronic fibrous cap. Eventually, after attempting sharp recanalization with the wire's back end, the wire was successfully passed through the hepatic venous end of the TIPS stent into the IVC. At this point, using a 15 mm gooseneck snare placed via the right internal jugular vein access, the .035-inch stiff Glidewire floppy end was snared and externalized through the transjugular access. By applying this “flossing” technique, through and through systemic venous and portal access was secured. (Fig. 4)

**Fig. 5:**
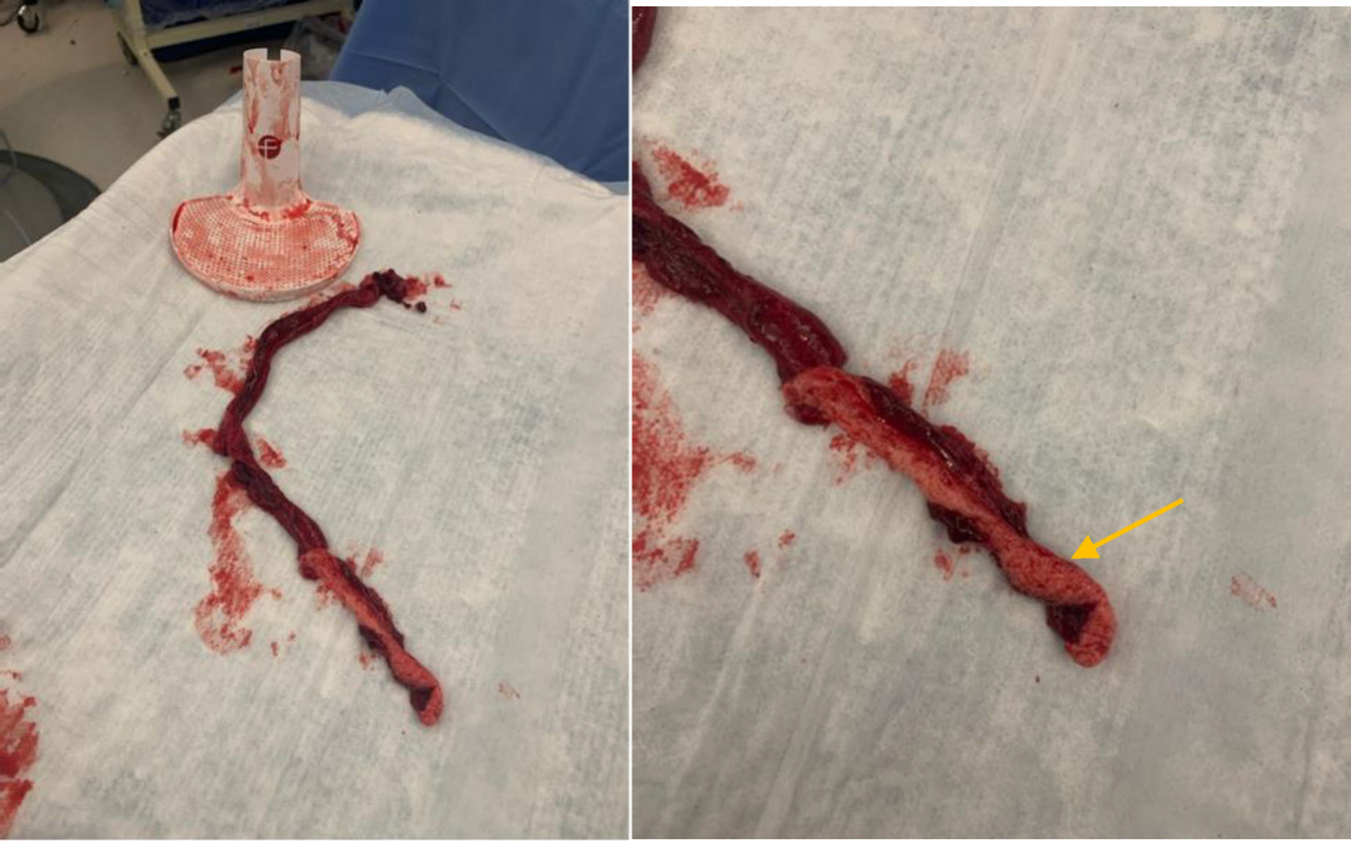
Large thrombus measuring 31 cm in length removed from TIPS stent with the Penumbra System Lighting12. The hepatic venous end fibrous cap can be seen (Orange arrow).

A 5-Fr MPB catheter was advanced into the TIPS stent over the .035-inch “flossing” Glidewire and after multiple attempts, the access to the portal vein through the stent was established after 3mm balloon dilatation. Given the extreme clot burden within the TIPS stent and the difficulty in securing and increasing the size of the access, the decision was made to proceed with mechanical thrombectomy. Thrombectomy was performed using the Indigo System Lightning 12 (Penumbra, Inc., Alameida, CL), and a well-formed clot measuring 31 cm in length, and 2-3 cm in thickness was removed. A well-formed fibrous cap was seen correlating with the stent's hepatic venous end (Fig. 5).

**Fig. 6:**
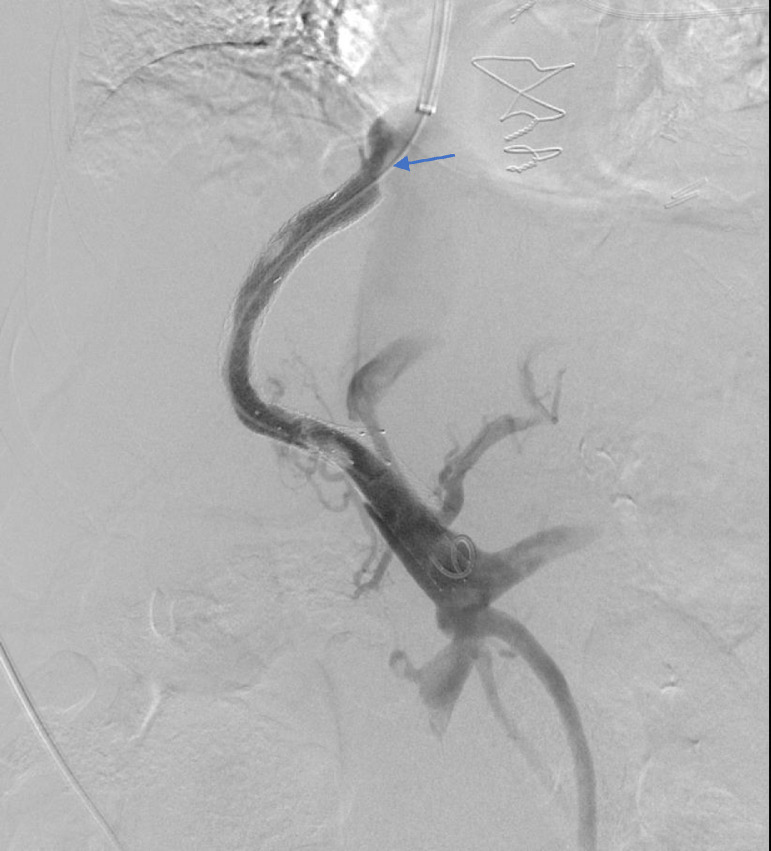
Final portal angiogram showing functioning TIPS shunt, with chronic mural based thrombus within the portal end and within the stent. Extension stent within the main portal vein to displace portal end of the malpositioned stent. Residual stenosis is seen despite angioplasty at the hepatic venous end (blue arrow).

The previously placed portal venous end extension stent was detached from the main TIPS stent and was completely occluded. Multiple attempts were made to re cannulate the occluded detached stent but were unsuccessful, as the stent did not appear to be in the expected location of the portal vein as seen in Fig. 2. Subsequently, we were able to pass the .035-inch stiff Glidewire through the main portal vein into the superior mesenteric vein. We then performed mechanical thrombectomy with the removal of multiple additional small clots. At this point, the decision was made to deploy an extension VICI venous stent (Boston Scientific, Marlborough, MA) measuring 12 mm by 60 mm to bridge the TIPS stent with the main portal vein and exclude the misplaced adjacent stent struts, followed by angioplasty with a 12 mm balloon of the entire stent complex. Direct portal pressure was measured at 38 mmHg. Repeat right atrial pressure was measured at 30 mmHg, resulting in a final portal-systemic gradient of 8 mmHg. Final portal angiogram showed a functioning TIPS shunt, with chronic mural based thrombus seen within the portal end and within the stent. Residual stenosis at the hepatic venous end was also seen (Fig. 6).

On follow-up one month later, the patient expressed marked symptomatic improvement and stated she felt significantly better. At this time, she was requiring one large-volume paracentesis per week, down from two per week prior to the procedure. Additionally, the removed volume decreased from 10 liters pre-TIPS revision to 3 liters post-TIPS revision. She was planned for follow-up portal venography and additional stent extension of the hepatic venous end given residual stenosis. Patient was then lost to follow-up.

## Discussion

Shunt stenosis or occlusion is a common complication after TIPS procedures with meta-analysis showing that reintervention to maintain shunt patency is required in 70%-90% of patients within 2 years of creation [3]. This data reflects the clinical scenario in our case as the patient underwent initial revision 13 months post-TIPS and the second revision described here 17 months post-TIPS. Thrombosis is another complication associated with TIPS, most commonly affecting the shunt, portal vein, or splenic vein in up to 12% of patients following the procedure. When thrombosis does occur, it typically presents within the first month after the procedure. Similarly, to stenosis, the incidence of TIPS thrombosis has also decreased considerably following the widespread adoption of PTFE-covered stents [2]. The higher rate of thrombosis associated with bare metal stents is thought to be related to the development of biliary-venous fistulas and thrombogenicity of bile [7].

Given the widespread use of covered stents, most cases of TIPS thrombosis are commonly caused by structural obstruction of flow related to suboptimal shunt position or configuration, which can occur due to initial malpositioning or migration of the shunt following the initial procedure. Malpositioned TIPS stents at risk for thrombosis are more commonly related to the hepatic venous end, with the highest risk related to the superior margin terminating within the hepatic parenchymal tract abutting the hepatic vein or IVC wall. Although initial portal venography after initial shunt insertion may show appropriate stent position and flow, removal of the guidewire or catheter can cause changes in stent positioning resulting in compromised flow through the TIPS. In our patient, thrombosis was attributed to the need for placement of several hepatic venous end extension stents and a non-anatomical non-overlapping portal venous end extension stent.

Treatment options for TIPS thrombosis include thrombectomy, balloon angioplasty, catheter-directed pharmacologic thrombolysis, systemic anticoagulation, or definitive treatment with liver transplantation. In most cases, a combined approach with thrombectomy and catheter-directed pharmacologic thrombolysis is used. Thrombectomy performed for management of TIPS thrombosis can be performed through multiple approaches – mechanical, hydrodynamic, and rheolytic methods have all been described [8]. Other non-traditional methods that have been described to successfully treat TIPS thrombosis include radiofrequency wire recanalization [9] and ultrasound-assisted thrombolysis [10]. If thrombosis is related to suboptimal positioning as described above, attempts can be made to cannulate and extend the existing TIPS with an additional stent, with creation of a new TIPS being required if this is not possible. If the malpositioning is related to the stent graft abutting the IVC, removal of the stent graft with a snare can be considered. Data supporting the use of systemic anticoagulation for prophylaxis or treatment of TIPS thrombosis is limited given that these patients are at high risk of bleeding.

Most cases of in-stent occlusion in TIPS can be managed via the transjugular approach with recanalization or placement of additional stents. In our patient, this approach could not be used to cannulate the occluded stent given the extent of well-organized clot located near the hepatic venous end after prolonged duration of stent occlusion of 13 months. As a result, a combined transjugular and transhepatic approach was used, which was first reported by Haskal and Cope in a series of 4 patients (technical success in 4/4) [11]. Tanaka et al. [12] and Chen et al. [13] have also described the successful use of this technique in cohorts of 2 patients (technical success in 2/2) and 14 patients (technical success in 13/14) with TIPS dysfunction. The primary patency time reported in the 14-patient cohort was 10.4 ± 2.9 months (range, 7-18 months). In our case, we encountered an extremely challenging scenario with 2 overlapping covered stents and a bare metal stent at the hepatic venous in the a setting of chronic thrombosis and a well-organized fibrous cap. The case highlights the need for optimal initial placement of the primary TIPS shunt in the first place to avoid the need for complex interventions to maintain TIPS shunt patency.

## Conclusion

In summary, stent thrombosis and occlusion are complications of TIPS procedures that present with recurrent symptoms of portal hypertension, which in our case was an increase in paracentesis burden. TIPS thrombosis can occur due to structural obstruction of flow related to suboptimal TIPS shunt initial placement or configuration, which can occur due to initial malpositioning or migration of the shunt following the initial procedure. Treatment options for TIPS occlusion involve recanalization or placement of additional stents, which in our case was performed via a combined transjugular and transhepatic approach. For patients with extensive thrombosis of the shunt, combined treatment with mechanical thrombectomy and catheter-directed thrombolysis may be needed.

## Patient consent

Written, informed consent was obtained from the patient for all procedures performed in this study.
